# Protein kinase substrate identification on functional protein arrays

**DOI:** 10.1186/1472-6750-8-22

**Published:** 2008-02-28

**Authors:** Lihao Meng, Gregory A Michaud, Janie S Merkel, Fang Zhou, Jing Huang, Dawn R Mattoon, Barry Schweitzer

**Affiliations:** 1 Invitrogen Corp., Protein Array Center, 688 East Main Street, Branford, CT 06405, USA; 2 Novartis Institutes for Biomedical Research, Inc., Developmental & Molecular Pathways, 250 Massachusetts Avenue, Cambridge, MA 02139, USA; 3 Yale University, Department of Biology, Kline Biology Tower, PO BOX 208103, New Haven, CT 06520-8103, USA; 4 Merck & Co., Inc., UG2C-24, 351 N. Sumneytown Pike, North Wales, PA 19454-2505, USA; 5 UCLA, Department of Molecular and Medical Pharmacology, 23-231 CHS, 10833 Le Conte Avenue, Los Angeles, CA 9009, USA

## Abstract

**Background:**

Over the last decade, kinases have emerged as attractive therapeutic targets for a number of different diseases, and numerous high throughput screening efforts in the pharmaceutical community are directed towards discovery of compounds that regulate kinase function. The emerging utility of systems biology approaches has necessitated the development of multiplex tools suitable for proteomic-scale experiments to replace lower throughput technologies such as mass spectroscopy for the study of protein phosphorylation. Recently, a new approach for identifying substrates of protein kinases has applied the miniaturized format of functional protein arrays to characterize phosphorylation for thousands of candidate protein substrates in a single experiment. This method involves the addition of protein kinases in solution to arrays of immobilized proteins to identify substrates using highly sensitive radioactive detection and hit identification algorithms.

**Results:**

To date, the factors required for optimal performance of protein array-based kinase substrate identification have not been described. In the current study, we have carried out a detailed characterization of the protein array-based method for kinase substrate identification, including an examination of the effects of time, buffer compositions, and protein concentration on the results. The protein array approach was compared to standard solution-based assays for assessing substrate phosphorylation, and a correlation of greater than 80% was observed. The results presented here demonstrate how novel substrates for protein kinases can be quickly identified from arrays containing thousands of human proteins to provide new clues to protein kinase function. In addition, a pooling-deconvolution strategy was developed and applied that enhances characterization of specific kinase-substrate relationships and decreases reagent consumption.

**Conclusion:**

Functional protein microarrays are an important new tool that enables multiplex analysis of protein phosphorylation, and thus can be utilized to identify novel kinase substrates. Integrating this technology with a systems biology approach to cell signalling will help uncover new layers in our understanding of this essential class of enzymes.

## Background

Eukaryotes have devoted approximately two percent of their genome to kinases, highlighting the importance of protein kinase function. Protein kinases are involved in numerous cellular processes, and aberrant kinase activity has been directly implicated in the etiology of a wide spectrum of human pathologies. In recent years, several kinase-directed drugs, including Gleevec^®^, Iressa^®^, Herceptin^® ^and Avastin^®^, were approved to treat human diseases [1]. Currently, more than 50 protein kinase drug candidates are in clinical trials to treat diseases including cancer, chronic inflammation, metabolic disorders, and neurodegenerative disease. The importance of protein phosphorylation in global regulation of cellular processes is apparent from estimates that at least one third of all proteins are phosphorylated [2]. For the vast majority of these proteins, however, the protein kinase(s) responsible for their phosphorylation is not known. In addition, the function of many protein kinases is completely unknown or has been poorly characterized. Despite their central role in health and disease, the identification of protein kinase substrates remains a significant challenge. Techniques that advance our knowledge about the substrates of specific kinases will certainly aid in our understanding of this biologically essential class of protein enzymes.

A growing number of methods to identify substrates of protein kinases are available. Most commonly, proteins from cells are isolated either from gels, by immunoprecipitation, or by metal affinity chromatography, and the phosphorylated sequences are determined by mass spectrometry [3-5]. Kinase-substrate relationships have also been determined through the use of enzyme inhibitors, functional knockouts and analogue sensitive kinase alleles combined with mass spectrometry. There are limitations inherent to these approaches, however, such as functional redundancy, poor characterization of enzyme-inhibitor specificity, and lack of sensitivity due to under-representation of low abundance proteins in these screens. In addition, mass spectrometry-based approaches often require considerable amounts of time and a high level of technical expertise to complete assays, operate instrumentation, and perform data analysis. The use of phospho-specific antibodies against consensus phosphorylation sites has been helpful in addressing some of these issues, but the consensus sequence information or high quality phospho-specific antibodies are not available for many discovery efforts.

*In vitro *based platforms using purified components offer solutions to some of the limitations of mass spectrometry-based approaches for protein kinase substrate identification. For example, the use of peptide arrays in substrate screens has been valuable for defining consensus phosphorylation sites for many protein kinases. However, it is very difficult to predict which cellular proteins are in fact substrates of protein kinases based solely on short primary sequence information, and using this data to identify *bona fide *kinase substrates is often problematic. Experiments have demonstrated that the ability of a kinase to modify a protein at a specific site is influenced by its structural context, including post-translational modifications such as glycosylation [6-8], localized sequential "priming" phosphorylations [9], inhibitory sequences [10], secondary structure and solvent accessibility [11]. For these reasons, using arrays of full-length but denatured proteins to screen for kinase substrates, as recently described by Feilner et al using arrays of denatured Arabidopsis proteins [12], has significant limitations, Solution-based assays using purified proteins that have been expressed, purified, and demonstrate functional activity can overcome some of these limitations but can be costly to achieve on a large scale.

Proof-of-principal for the use of arrays of native or functional proteins for kinase substrate profiling was described by MacBeath and Schreiber [13]. Although this study utilized arrays of only a few proteins, it demonstrated some of the advantages that functional protein arrays offer over existing assays. For example, arrays containing thousands of proteins can be prepared with similar amounts of protein to rapidly establish kinase-substrate relationships with limited concentration-based artefacts. Second, the identity of the substrates is known immediately following the assay since full-length proteins are used and the position of each protein on the array is known. Third, the probability that a phosphorylated structured protein revealed in an array experiment is indeed a cellular target of the kinase should be much higher than candidate substrates identified from experiments based on peptides. Lastly, because arrays may contain up to thousands of proteins, target selectivity is quickly addressed with minimal amounts of reagent. Recently, a seminal study by Ptacek *et al*. profiled the activity for 87 purified yeast protein kinases against a proteome array containing over 4000 proteins expressed and purified from *S. cerevisiae *[14]. The approach successfully identified thousands (4,192) of phosphorylation events mapping to 1,325 different substrate proteins. The integration of these results with multiple data types has also led to new proposals regarding yeast protein kinase interaction networks. More recently, Boyle et al. used high-content human protein microarrays to identify the actin-regulatory protein cortactin, a protein that is upregulated in several cancers, as a novel substrate of the Abl and Abl-related gene (Arg) nonreceptor tyrosine kinases [15]. These investigators went on to show that Abl-family kinases target cortactin as an effector of cytoskeletal rearrangements in response to PDGF.

In the current study, a detailed characterization of the protein microarray kinase-substrate identification assay is presented. Arrays of human proteins are used, and critical experimental parameters are investigated to address optimal assay performance. We show that the kinase-substrate phosphorylation microarray assay reconstitutes enzyme-substrate interactions observed in solution-based assays using a set of solution-validated protein kinase-substrate pairs. Results are also presented that show that these arrays can be used to quickly identify novel substrates of human protein kinases on functional human protein arrays containing thousands of highly purified proteins and that pooling-deconvolution strategies can be employed to rapidly identify specific kinase-substrate relationships.

## Results and Discussion

### Solution validation of kinase-substrate interactions

Measuring phosphorylation of proteins by kinases in solution-based assays may be the most common means of confirming that a protein is indeed a substrate for a particular kinase. Consequently, substrate phosphorylation assays using predominantly full-length proteins on protein arrays were compared to results generated in solution assays for a number of commercially available kinase-substrate pairs in order to determine the accuracy of the array-based method. Kinase-substrate reactions were first performed in solution to confirm that the protein kinases indeed phosphorylated their intended substrates. This involved mixing the protein kinases with the substrate proteins in the presence of ^33^Pγ-ATP, incubating the samples at 30°C, resolving the proteins on SDS-page gels and using a phosphorimager to detect substrate phosphorylation in the presence of kinase. A panel of 24 kinase-substrate interactions comprising 14 different protein kinases and 18 substrates was defined as the test set to evaluate the ability to replicate the protein substrate phosphorylation observed in solution on protein arrays (Table 1).

**Table 1:** Kinase-substrate pairs.

		**Solution Assay**	**Protein Array Assay**	
			**Z_Score**	
			
**Upstream Kinases**	**Substrates**	**(Signal/Background)**	**-BSA**	**+BSA**

CaMKII	Tau Protein	16.01	15.00	10.1
CDK5/p35	Tau Protein	28.24	18.80	6.5
CK2	pTEN	67.65	12.80	18.6
ERK2(MAPK)	4EBP/PHAS-1	3.85	6.10	9.6
ERK2(MAPK)	Elk-1 Fusion Protein	8.17	17.00	20
ERK2(MAPK)	Tau Protein	3.34	24.10	19.4
GSK3beta	GST-Axin	33.63	1.40	2.4
JNK1	c-Jun 1–79	6.84	19.20	103.3
MAP2K6(MKK6)	MAPK12 Inactive	20.45	0.90	0.1
MAP2K6(MKK6)	MAPK14, Inactive	42.02	12.50	23.6
MAPK14 (p38alpha)	4EBP/PHAS-1	3.58	9.90	17.5
MAPK14 (p38alpha)	ATF2 (aa 19–96)	6.48	22.60	25.5
MAPK14 (p38alpha)	MAPKAP-K2 Inactive	13	13.90	19.4
MAPK14 (p38alpha)	MAPKAP-K3 Inactive	6.21	10.70	22.4
MAPK14 (p38alpha)	MAPKAP-K5, Inactive	5.07	5.40	2.4
MKK4/SKK1	MAPK12 Inactive	17.43	1.80	7.7
MKK4/SKK1	MAPK8 (JNK1) Inactive	23.17	2.90	0.7
MKK4/SKK1	MAPK9 (JNK2) Inactive	6.61	0.90	5.9
p38β2/SAPK2b2	MAPKAP-K3 Inactive	12.63	3.40	9.4
PDK1	AKT2 Inactive	6.33	4.30	3.1
PKA	ATF2 (aa 19–96)	8.34	3.40	0.7
PKA	Tau Protein	12.66	33.90	4.6
ROKα/ROCK-II	MYPT1 (654–880)	51.54	14.40	23
RPS6KA3(RSK2)	Estrogen Receptor-α	9.08	0.40	2.7

A set of 24 kinase-substrate interactions comprising 14 different protein kinases and 18 substrates was defined as the test set for benchmarking protein microarray results against solution phase assays. Solution phase reactions were run for each of these pairs in the presence of ^33^P-ATP. After 1 hour, reactions were terminated by the addition of sample buffer and run on SDS-PAGE gels. Gels were subsequently dried, exposed to a phosphoscreen, and imaged in a Cyclone phosphoimager. Pixel intensity data from the resultant high resolution images was extracted and used to calculate the reported signal/background values. Protein microarray assays were run by incubating arrays with exogenous kinase in the presence of ^33^P-ATP. After 60 minutes, arrays were washed, dried, exposed to a phosphoscreen, and imaged using a Cyclone phosphoimager. Pixel intensity data from the resultant high resolution images was extracted using GenePix software, and used to calculate the reported Z-Scores.

### Kinase-substrate interactions on protein arrays

**Figure 1:**
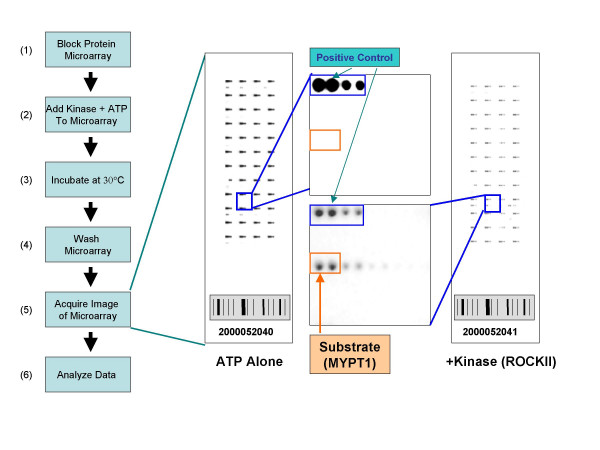
**Microarray kinase substrate identification assay**. A schematic of the experimental procedure used for kinase substrate identification on ProtoArray^® ^is shown on the left. Representative slide images and enlarged regions show autophosphorylation of printed landmark kinase spots and a specific example of substrate phosphorylation for ROCKII kinase.

**Figure 2:**
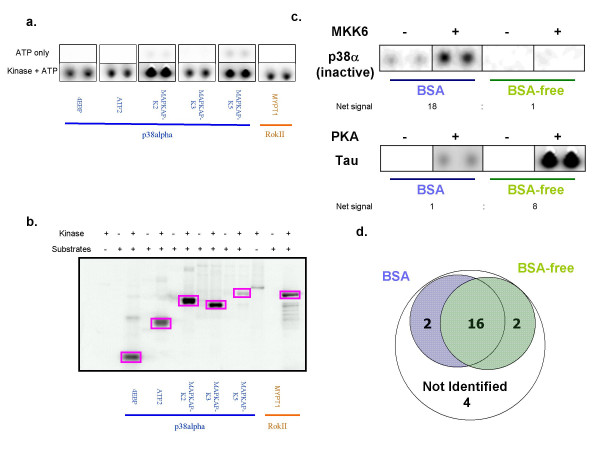
**Substrate phosphorylation by protein kinases on ProtoArray^®^**. (a) Examples of substrate phosphorylations on protein microarrays in the presence of p38alpha and ROKII kinases. (b) Solution validation of commercial kinase substrate pairs. Square box indicates substrate phosphorylation by corresponding kinase. (c) Comparison of different kinase buffers on substrate phosphorylation., Phosphorylation of inactive p38alpha by MKK6 was observed only in Buffer system I (BSA). Tau protein was phosphorylated by PKA to a greater degree in Buffer System II (BSA-free). Net signal was determined by calculating a ratio for the [Kinase-ATP only]^BufferI^/[Kinase-ATP only]^BufferII ^(d) Venn diagram of substrates phosphorylated by kinases in different buffer systems.

Eighteen protein substrates were printed on modified glass slides using a standard contact-type quill pin arrayer. Purified protein kinases were added to the arrays in the presence of ^33^Pγ-ATP, incubated at 30°C for different periods of time and rigorously washed to remove free kinase and ATP. Images and data were acquired using a phosphorimager and standard microarray data acquisition software (Figure 1). Protein kinases immobilized at defined locations on the array facilitated mapping of substrates by defining reference spots within the array image (Figure 1, positive controls). When ^33^Pγ-ATP is added to the arrays, these protein kinases autophosphorylate on the surface and generate a radioactive signal upon detection. An example of a protein kinase-substrate interaction is shown in Figure 1. The Rho-associated protein kinases are known upstream regulators of myosin phosphatase target subunit 1 (MYPT1) and control smooth muscle contractility by negatively regulating its activity [16,17]. Significant signal for MYPT1 is observed only in the presence of Rho-associated coiled coil containing protein kinase 2 (ROCKII). No signal is observed for MYPT1 on the negative control slide (ATP alone). The performance of the 24 kinase-substrate pairs was assessed in a similar fashion on protein arrays using two protocols that were distinguished primarily by the absence or presence of bovine serum albumin (BSA) included in the blocking and probing buffers. Eighteen kinase-substrate interactions were observed using a protocol that included BSA (slide blocking and kinase incubation steps both contain BSA). Images of substrates phosphorylated by p38α and ROCKII are shown in Figure 2a. Note that significant signals were observed only in the presence of kinase (ATP only versus Kinase + ATP). This is consistent with the results of the solution assays for these same kinase-substrate pairs (Figure 2b).

Six of the kinase-substrate interactions in the test set were not observed on the arrays using the standard protocol when a static Z-Score threshold of 3.0 was applied, where the Z-Score represents the number of standard deviations above the median signal value for all protein features present on the array. BSA is included at a relatively high concentration (10 mg/ml) in the standard array kinase-substrate assay in order to block non-specific interactions, but was not included in the solution phase assays used to develop the test set. Eighteen kinase-substrate interactions were observed using an array assay protocol that did not contain any BSA, with 16 interactions overlapping with those observed in the BSA protocol when a Z-Score threshold of ≥3.0 was applied (Table 1). Several kinase-substrate interactions exhibited significantly different signals depending on the protocol that was used. For example, the signals for Protein Kinase A (PKA) phosphorylation of Tau protein were approximately eight-fold greater in the absence of BSA (Figure 2c). Conversely, the signal for Map Kinase Kinase 6 (MKK6) phosphorylation of p38α was approximately 18-fold greater in the presence of BSA (Figure 2c). After combining the results of both protocols, a total of 20 true positives out of a possible 24 (83%) kinase-substrate interactions could be observed on protein arrays.

### Effect of array protein concentration

**Figure 3:**
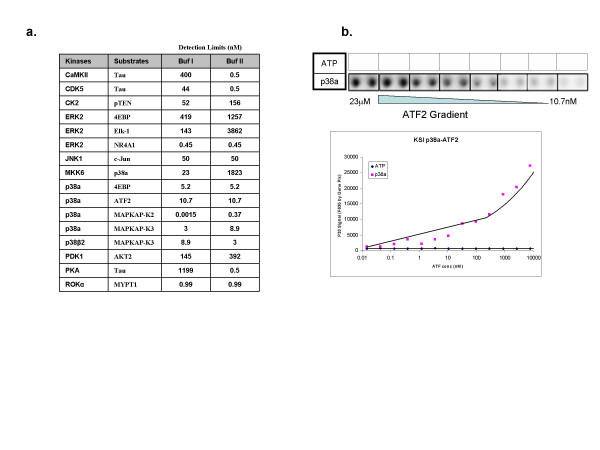
**Assay sensitivity**. (a) The limit of detection (LOD) of each kinase-substrate pair in both buffer systems using ≥ = 3× STDEV of the negative control (GST) as a threshold. (b) Images and quantitation of ATF2 by p38alpha.

In the high throughput process employed to generate thousands of proteins for the high-density protein microarrays, different proteins can vary up to two logs in the amount of protein that gets deposited on the array. In order to examine the effect of this variation on the sensitivity of the kinase substrate profiling assay, protein substrates were spotted in a gradient of concentrations from a maximal solution concentration of approximately 10 μM to a minimum of 10 pM. Kinase-substrate assays were performed with the 16 protein kinase substrates using both the BSA and BSA-free protocols. A lower limit of detection was calculated for each kinase-substrate interaction for each protocol by defining the minimum amount of protein (relative solution concentration) spotted that is required to observe significant signals on the arrays. For 10 of the 16 kinase-substrate interactions, the lower limits of detection varied only four-fold when comparing the BSA and BSA-free protocols (Figure 3a). Figure 3b shows an example of the protein concentration dependent phosphorylation of ATF2 by p38 alpha. The lower limit of detection for the p38 alpha-ATF2 interaction was estimated to be about 10 nM. Interestingly, for six kinase-substrate interaction pairs, the observed lower limits of detection differed by 30–2000-fold depending on whether BSA was present in the assay. For example, the lower limit of detection for the phosphorylation of Tau by PKA was approximately 2 μM in the presence of BSA, but was only 0.5 nM when the BSA-free protocol was employed. In contrast, the lower limit of detection for the phosphorylation of p38 alpha was 2 μM for the BSA-free protocol, yet only 23 nM in the presence of BSA. Thus, both the amount of protein spotted and the presence of BSA in the assay can influence the sensitivity of the assay and the ability to observe substrate phosphorylation.

### Effect of Time and ATP Concentration

**Figure 4:**
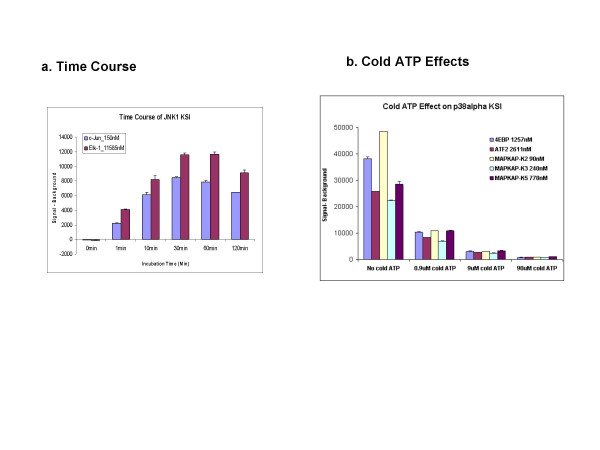
**Effect of time and ATP concentration on assay performance**. (a)Time course of phosphorylation observed for two substrates of JNK1 kinase. The net P^33 ^signals were plotted. (b) Different amounts of non radioactive ATP (cATP) were added to the reaction mixture and compared to assays with a constant amount of radioactive ATP (γ^33^P-ATP). The P^33 ^signals (average from two slides) of different substrates are compared for different amounts of cATP.

It was of interest to determine whether the sensitivity of the assay could be further improved by varying other assay conditions. Two protein kinases, JNK1 and p38 alpha (MKK14) (Table 1), were incubated on arrays at 30°C for 0, 1, 10, 30, 60 or 120 minute prior to washing and detection. As shown in Figure 4a, signals for each kinase-substrate interaction were greatest on average at 30–60 minutes following addition of the kinase. At 120 minutes, net phosphorylation signals of c-Jun and Elk-1 by JNK1 were decreased relative to the 30 and 60 minute time points due to an increase in background on the protein array (Figure 4a).

The protein microarray assay exclusively uses radiolabeled ATP (^33^Pγ-ATP) at a relatively low concentration (33 nM) compared to most kinase-substrate phosphorylation assays, which typically utilize micromolar quantities of ATP. The K_M _for most protein kinases is approximately 50 micromolar, raising the possibility that the enzymatic efficiency of the array assays at 33 nM ATP may not be optimal. However, when 0.45, 4.5 or 45 μM unlableled ATP was added to the p38 alpha kinase array assay, thereby providing approximately a 10-, 100-, or 1000-fold excess of unlabeled ATP, a concentration-dependent decrease in signal was observed for both the positive control protein kinases and the substrates (Figure 4b).

### Assay Reproducibility

**Figure 5:**
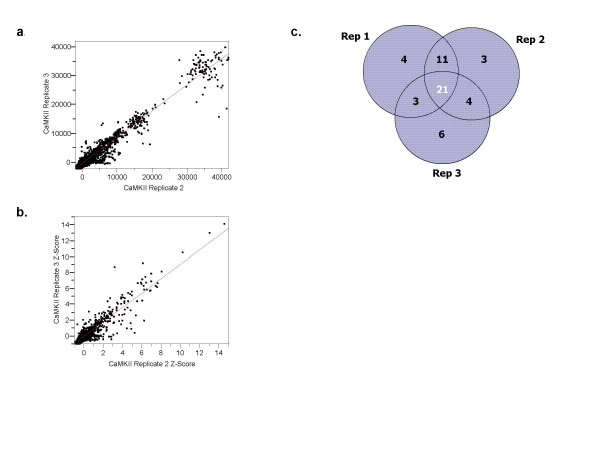
**Identification of CAMK2 substrates on high content Human ProtoArray^®^**. (a and b) Scatterplots showing reproducibility in replicate assays. (c) Venn overlap of CAMK2 substrates identified from triplicate assays.

The reproducibility of the method was determined from triplicate kinase assays of 50 nM CaMKII in the presence of the cofactors calcium and calmodulin on protein microarrays containing 3019 different human proteins printed in duplicate and a total of 6244 accessory features (control proteins and buffer/no protein spots). BSA was included in the assay as described above. Pairwise comparisons of background-corrected protein spot signals (n = 6044) were linearly correlated throughout the signal range with slopes ranging from 1.17 – 0.90, and with R^2 ^correlation coefficients calculated between 0.92 and 0.96 (Figure 5a). Background-corrected human protein spot signals were averaged for duplicate spots on a single array, and Z-Score transformed such that the distribution of human protein spots within an assay was centered with a mean of zero and a standard deviation of 1. Thus, a Z-Score of 3 indicates a phosphorylation signal exactly three standard deviations above the mean value for the human protein signals. In this experiment, phosphorylation of a human protein by CaMKII was considered significant if the human protein Z-Score was equal or greater than 3, the duplicate spots coefficient of variation less than 0.5, and the background-corrected signal in a CaMKII assay was at least 150% of the background-corrected signal in the no kinase control assay. Fifty-two human proteins were found to be significant in at least one of the three CaMKII assays, but not in the CaMKII-free control assay (Figure 5b). In general, proteins significant in all three CaMKII assays had the highest signals, followed by proteins present in two assays, and then by proteins significant in a single assay. For 94% of the proteins identified, the Z-score in the no kinase control assay was below 1. Among the 52 proteins, 32 were expressed and purified for validation in solution-based assays. Twenty-three of the 32 putative substrates identified on the array were also phosphorylated by CaMKII in solution (See Additional file 1).

### Substrate Identification on High Content Protein Arrays

**Figure 6:**
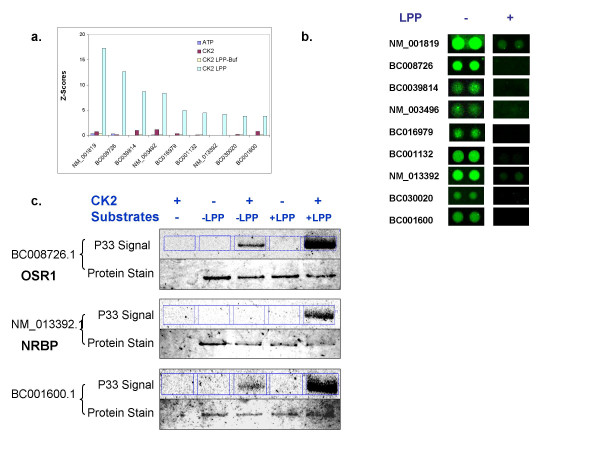
**Effect of phosphatase pre-treatment on kinase phosphorylation of proteins on microarrays**. (a) CK2 substrates identified on phosphatase pre-treated high-content Human ProtoArray^® ^4. (b) Decrease in phosphate levels of protein microarrays treated with lambda protein phosphatase (LPP) followed by staining with Pro-Q Diamond. (c) Solution validation of CK2 substrates identified after LPP treatment.

The yeast proteome microarrays used to map the yeast phosphorylome were generated through a process involving high throughput protein expression and purification using affinity chromatography. The output of this process is thousands of functional proteins that are greater than 90% pure (data not shown). More recently, this process has been applied to commercial protein microarray products (ProtoArray^® ^technology) containing thousands of individually purified human proteins [14]. Many of the proteins used to manufacture the yeast and human protein microarrays (See Methods) are phosphorylated during expression (data not shown). This raises the possibility that the endogenous protein phosphorylation could interfere with protein kinase substrate phosphorylation on protein arrays by masking phosphorylation sites. To investigate this further, proteins on the array were dephosphorylated using a general protein phosphatase and then incubated with Casein Kinase II (CK2). The level of phosphoprotein content on the protein arrays was monitored by probing the arrays with a fluorescent stain (Pro-Q Diamond) that binds to phosphoamino acids (Figure 6) [18]. Interestingly, nine proteins on a ProtoArray^® ^Human Protein Microarray v1.0 containing over 2,000 human proteins produced significant signals only after phosphatase treatment (Figure 6a). Seventeen proteins produced significant signals without phosphatase treatment. The substrate phosphorylation observed on the array was validated by performing solution assays which demonstrated that dephosphorylation of the substrates resulted in enhanced levels of protein phosphorylation for OSR1 [GenBank: BC008726.1], NRBP [RefSeq: NM_013392.1] and C10orf7 [GenBank: BC001600.1] (Figure 6c). Both OSR1 and NRBP are known phosphoproteins [19,20].

### A pooling-deconvolution strategy for substrate identification

Recently, an approach was described to improve the efficiency and accuracy of large scale screening experiments in which pools of potential interactors are applied against a defined library and then an algorithm is used to deconvolute the results [21]. This strategy was applied to yeast two-hybrid and small molecule/cell survival screens and subsequently validated using protein arrays. In the validation study, protein-protein interaction assays were performed using pools of proteins probed against yeast protein microarrays comprised of more than 4000 yeast proteins, followed by successful deconvolution of expected individual interaction pairs.

**Figure 7:**
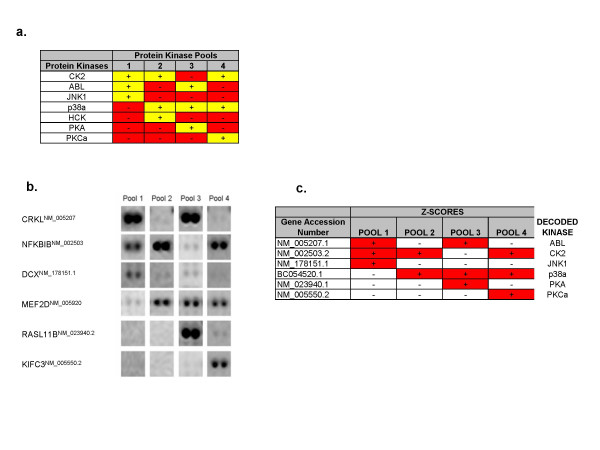
**Use of Pooling-Deconvolution for kinase substrate identification on protein microarrays**. (a) Composition of kinase pools used in assays (b) Representative images of substrate phosphorylations on arrays show how the pooling scheme can be used to assign specific substrates to their corresponding kinases. (c) Decoding of kinases responsible for substrate phosphorylation.

The results presented here extend this work by demonstrating that a pooling/deconvolution strategy can also be used with protein kinases to identify specific protein kinase substrates on high content protein arrays. Four unique pools of protein kinases were prepared (Figure 7a) that contained up to three protein kinases in each pool. Each pooled mixture of kinases was then added separately to a high content human protein microarray. Multiple phosphorylation events were observed on each array (data not shown). The signals for six of the phosphorylated proteins are shown in Figure 7b. These images show that the signal for each protein substrate varied depending on the kinase pool added to the array; for example, significant signals were only observed for CRKL in pools 1 and 3 (Figure 7b/c). When the pattern of significant signals was compared to the composition of protein kinases in the pools, it could be seen that Abl was the only protein kinase that is present in pools 1 and 3 and absent in pools 2 and 4; thus allowing definitive identification of Abl as the kinase responsible for CRKL phosphorylation. The same process was applied to the other substrates to identify specific kinase-substrate interactions for 6 of the 7 protein kinases (Figure 7c). The decoded protein kinase-substrate interactions were consistent with data obtained from arrays treated with single protein kinases (See Additional file 2).

## Conclusion

Posttranslational modification of proteins is one of the principle regulatory mechanisms in eukaryotes. In particular, protein phosphorylation has been demonstrated to be crucial in the proper regulation of nearly all cellular processes, including metabolism, cell organization and differentiation, responses to environmental stimuli and cell-cell interactions. Nevertheless, a thorough understanding of the full range of phosphoproteins modified by a specific protein kinase is often lacking. Proteome microarrays have been described in which sets of proteins, or nearly every protein in the case of yeast, were expressed, purified, and deposited on a surface in an addressable format [22-24]. Using such a proteome-scale microarray, Ptacek et al created a map of the yeast phosphorylome by incubating the arrays with nearly every yeast kinase and identifying thousands of phosphorylation events [14]. These studies revealed a plethora of potentially novel biological functions as well as new regulatory interactions for a spectrum of kinases. More recently, a human protein array was used to identify a novel substrate for Arg and Abl kinases, the targets of the anti-cancer drug Gleevec [15]. In the present study, we performed a detailed characterization of the ProtoArray^® ^technology as employed to define kinase-substrate interactions, and have identified a number of key features that can impact kinase-substrate discovery efforts.

The first task was to benchmark performance of the kinase-substrate application on protein arrays relative to standard solution-based assays. This was accomplished by defining a test set of kinase-substrate pairs and determining the correlation of substrate phosphorylation on the array relative to a solution assay. Twenty out of 24 (83%) kinase-substrate pairs were reconstituted on arrays, demonstrating that substrate phosphorylation on protein arrays is highly consistent with solution-based methods, even under the relatively generic conditions employed in these assays. Several possibilities exist for the inability to observe phosphorylation of four protein on the arrays including (1) binding of the protein to the array surface in such as way as to preferentially mask a phosphorylation site; (2) denaturing of the protein on the array that abolishes a kinase interaction domain; (3) altered kinetics of the phosphorylation reaction on the array surface; or (4) a combination of several of these factors. Nevertheless, this "false-negative" rate must be considered in light of the advantages of the array format including speed, low material requirements, and the ability to survey thousands of purified proteins in a single experiment.

In a second set of experiments, we investigated the effect of assay conditions – specifically, the presence or absence of BSA in the assay buffer – on the results obtained with the arrays. Interestingly, 75% of the proteins phosphorylated in solution were observed to be phosphorylated on the arrays either in the presence or absence of BSA; however, the substrates identified under the different conditions did not completely overlap. A subset of protein substrates were observed only in the presence of BSA, while others were only seen to be phosphorylated on the array in the absence of BSA. In the former case, BSA may be stimulating substrate phosphorylation through a 'crowding' effect by increasing the effective local concentration of protein kinase at the array surface. In the latter case, BSA may be interfering with substrate phosphorylation through either binding to protein kinases, and/or by being a substrate for the kinase itself, thereby lowering the amount of available ^33^Pγ-ATP and/or kinase in the assay below the threshold required to give adequate phosphorylation of the substrate. Assays in which BSA was included in the buffer gave rise to a higher mean Z-Score across the test set of 24 kinase-substrate pairs, with 17 of the 24 pairs resulting in higher Z-Scores when BSA was present in the assay buffer. Based on this result, BSA-containing buffers are recommended if a single assay condition is to be employed. Further investigation will be required to determine the exact mechanism of the effect of BSA in this assay, and carrying out assays under both assay conditions should be considered in order to maximize the probability of observing substrate phosphorylation.

The study investigating the yeast phosphorylome reported that approximately two-thirds of previously annotated phosphorylation events were not observed in their protein array experiments. One possible explanation for this observation is that the amount of protein spotted on the array was too low to be phosphorylated by kinase. To explore this issue, we measured the relative amount of protein that must be spotted on the arrays for significant phosphorylation to be observed, and determined that the median LOD for the test set of kinase-substrate interactors was approximately 2 ng/μL, which is equivalent to approximately 2 pg of spotted protein. The median level of proteins on the yeast and human proteome arrays are approximately four-fold greater than this amount, suggesting that the majority of proteins on the arrays are present in quantities sufficient to observe phosphorylation. It should also be noted that this amount of spotted protein is approximately 1000-fold less protein than is required to observe phosphorylation in a solution-based assay.

Another factor that could influence the identification of proteins on the array as substrates for exogenous kinases could be masking of potential phosphorylation sites as a result of phosphorylation occurring during protein production by endogenous kinases. Using both anti-phosphoamino acid antibodies and phosphoamino acid stains such as ProQ Diamond, we have shown that many yeast and human proteins expressed from either yeast or insect cells and spotted on protein arrays are indeed phosphorylated (data not shown). As shown in Figure 6, CK2 phosphorylated several proteins only after dephosphorylation of proteins on the array with lambda protein phosphatase. For some proteins, the removal of the phosphate groups through enzymatic treatment with phosphatase was required in order to observe substrate phosphorylation on the array. It should also be pointed out that phosphorylation by some kinases may require that substrates are pre-phosphorylated or "primed" on specific residues.

We have provided further evidence that high content human protein arrays can be used to make novel insights into the biology of human protein kinases. In one example, 23 substrates were identified and validated for CamK2, 11 of which could be assigned to specific functions or pathways. The two protein substrates exhibiting the strongest signals were Doublecortin (DCX) transcript variants 4 and 2. Doublecortin, which has not previously been reported as a CAMK2 subsrate, is important for neurite outgrowth in the developing brain, and acts by stabilizing microtubules [25]. The observation that that calmodulin-kinase 2 phosphorylates doublecortin suggests a mechanism by which CamK2 regulates neuronal migration by influencing microtubule stability, and is consistent with the well established role for CamK2 in neuroplasticity [26,27].

We have also demonstrated for the first time that a pooling-deconvolution strategy can be applied to protein kinase substrate identification on protein arrays. In these experiments, four protein substrates were identified (FLJ22795, SH3YL1, CRKL, ABI1) that were uniquely phosphorylated on arrays treated with the Abl protein kinase. Signaling through Abl is critical to regulation of several cellular functions including organization of the actin cytoskeleton [28]. CRKL is a known substrate of Abl [29], and ABI1 is a known Abl interacting protein that has been shown to facilitate phosphorylation of Mena, a protein involved in cell adhesion and motility [30]. The protein SH3YL1 is homologous to the yeast protein Ysc84, which localizes to the cortical cytoskeleton in yeast and is involved in coupling endocytosis to the actin network [31]. This type of data argues that *bona fide *interactors can quickly be identified from screens on protein arrays. However, it is likely that a subset of proteins identified from such screens will not validate *in vivo *as protein kinase substrates, particularly if kinase and substrate occupy separate subcellular compartments. In addition, various factors in the in vitro array assay, such as kinase and/or substrate concentration, cofactors or lack thereof, or ionic conditions, may lead to inappropriate phosphorylation events. Finally, it is likely that many membrane-associated proteins, especially those which have regions that span the membrane, do not exhibit a native conformation on the array due to the lack of lipid, and thus may be phosphorylated in regions that are normally not accessible to a kinase. Assimilation of protein microarray data with orthogonal data types such as protein expression, localization, and interaction networks will most certainly enrich inventories of *in vitro *kinase-substrate pairs and expand our understanding of protein kinases function in cellular processes. We believe such integrated datasets will provide novel insights to intracellular phosphoprotein signaling which could ultimately foster important new efforts for drug discovery and development.

## Methods

### Protein Arrays

Commerical protein substrates were purchases from Cell Signalling, Upstate, Calbiochem and Invitrogen. Commercial protein kinases were purchased from Upstate and Invitrogen. All clones used to generate the human protein collection were fully sequenced and subcloned into the expression vector, pDEST™20 (Invitrogen Corp.). These clones were then used to express proteins in Sf9 insect cells as N-terminal GST-fusions using the Bac-to-Bac^® ^Baculovirus Expression System (Invitrogen Corp). Insect cell lysates were loaded directly into 96-well plates containing glutathione resin (GE Healthcare). After washing, proteins were eluted under non-denaturing conditions by the addition of 10 mM glutathione. ProtoArray^® ^Human Protein Microarrays v1.0 (Invitrogen Corp., Carlsbad, CA) were printed on 1 × 3 inch modified glass slides using a 48 pin contact arrayer (OmniGrid, Genomics Solutions). Protein arrays were stored at -20°C until use.

### Solution kinase assay

10 μl reaction mixtures contained assay buffer II (BSA-free) (1% Brij35, 100 mM MOPS pH 7.2, 100 mM NaCl, 5 mM MgCl_2_, 5 mM MnCl_2_, 1 mM DTT), ^33^Pγ-ATP (33 nM, 1 μCi/μl), substrate (10–100 ng), and kinase (1–50 nM). Assays were incubated at 30°C for 1 hour and terminated by the addition of 10 μl 2× SDS NuPAGE^® ^Sample Buffer. Proteins were denatured in a 95°C water bath for 10 minute. 20 μl of samples were loaded into NuPAGE^® ^10% precast gels. Electrophoresis was performed on the gels at 120 V for 1 hour. The gels were fixed in 10% acetic acid and 45% methanol for 45 minutes at RT, then washed twice with ddH_2_O for 30 minutes. Gels were encased in Saran Wrap and transferred to film cassette containing a phosphorimager screen to detect ^33^P activity at room temperature overnight.

### Kinase Substrate Identification (KSI) microarray assay

For Buffer System I (BSA), the blocking buffer was 1% BSA in PBS, and the kinase reaction buffer contained 1% BSA, 1% NP-40, 100 mM MOPS pH 7.2, 100 mM NaCl, 5 mM MgCl_2_, 5 mM MnCl_2_, 1 mM DTT. For Buffer System II (BSA-free), the blocking buffer contained 50 mM Tris pH 7.5, 0.1% Brij35, and 5 mM MgSO_4_, and the kinase reaction buffer contained 1% Brij35, 100 mM MOPS pH 7.2, 100 mM NaCl, 5 mM MgCl_2_, 5 mM MnCl_2_, 1 mM DTT. Protein microarrays were blocked for 2 hours in blocking buffer. 120 μl of kinase reaction mixture containing γ^33^P-ATP (1 μCi/μl) and kinase (1–50 nM) was added to the surface of the microarrays. Arrays were covered with a Hyperslip™ coverslip, placed into a 50 ml cubicle tube, and transferred to a 30°C incubator with the array face up. After 60 minutes incubation, arrays were washed twice with 0.5% SDS and then twice with distilled water. After washing, arrays were placed into a 25-slide holder and spun for 2 minutes at 2000 rpm in a plate centrifuge. Dry arrays were exposed overnight to a phosphorimager screen and images were analyzed by the Genepix software and further analyzed using Microsoft Excel software or Prospector (Invitrogen). Three independent samples of 50 nM CaMKII and 33 nM γ^33^P-ATP in Buffer System I were prepared and incubated on arrays, alongside a single negative control assay lacking kinase. A second set of three independent samples of 50 nM CaMKII and 33 nM γ^33^P-ATP in the presence of 5 mM CaCl_2 _and 600 nM calmodulin, in Buffer System I was prepared and incubated on arrays, alongside a single negative control assay lacking kinase. For pooling-deconvolution assays, kinase pools were prepared in Buffer System I.

### Phosphatase treatment of the protein microarray

The phosphatase reaction mix contained 400 Unit/ml of lambda protein phosphatase (New England Biolabs), 50 mM Tris-HCl pH7.5, 0.1 Na_2_EDTA, 5 mM DTT and 0.01% Brij35. 120 μl of phosphatase reaction mixture was added to the surface of the microarrays. Arrays were covered with a Hyperslip™ coverslip, placed into a 50 ml cubicle tube, and transferred to a 30°C incubator with the array face up for 2 hours. Arrays then proceeded to the KSI microarray assay or the Pro-Q microarray staining assay.

### Pro-Q microarray staining

Microarrays were blocked in 1% BSA, 50 mM HEPES pH 7.5, 200 mM NaCl, 0.1% Triton X-100, 25% glycerol, 20 mM reduced glutathione, 0.5 mM DTT at 4°C for 2 hours. Arrays were placed into a 25 slide holder and spun at 4000 rpm for 2 minutes to remove excess liquid from the slide surface. Immediately, 120 μl of Pro-Q Diamond™ phosphoprotein/phosphopeptide microarray stain was added to the surface of the microarrays. Arrays were covered with a Hyperslip™ coverslip, placed into a 50 ml cubicle tube, and incubated for 30°C at RT with array face up. Arrays were washed with Pro-Q Diamond microarray-destaining solution twice for 15 minutes and then washed with water twice for 15 minutes. After washing, arrays were placed into a 25-slide holder and spun 2 minutes at 2000 rpm in a plate centrifuge to dry. Dry arrays were scanned with an Axon Scanner using the 535 nm wavelength setting, 100% Laser Power, and 600 PMT. Images were analyzed by the Genepix software and further analyzed using Protoarray™ Prospector (Invitrogen).

### Data analysis

Low content arrays: Negative GST control signals were used to determine background signals for each subarray. For each spot, the mean background value was subtracted from the signal for spots in each subarray. Background-corrected signals were called significant when they were greater than three times the standard deviation of the average background signal in the same subarray. High content arrays: Arrays were analyzed in ProtoArray Prospector 3.0, including the signal scatter correction feature, and background signal normalization performed per subarray from the signal mean from GST and buffer spots.

## Authors' contributions

JM and FZ carried out the design of the arrays and the development of the basic assay protocol. LM carried out the time course and validation experiments as well as experiments examining the effects of BSA and phosphatase treatment. JH carried out the design and interpretation of the pooling/deconvolution experiments. GM participated in the design of the study, performed the statistical analysis, and helped to draft the manuscript. DM made intellectual contributions and participated in drafting the manuscript. BS conceived of the study, participated in its design and coordination and helped to edit the manuscript. All authors read and approved the final manuscript.

## Supplementary Material

Additional file 1 **CAMK II substrate identification**. CamK II assays were performed on Human ProtoArrays^® ^using three protocols – Buffer System I protocol, Buffer System II protocol, and lambda protein phosphatase-pretreatment protocol. Each assay condition was performed in triplicate, including the ATP negative controls. Substrates were identified by having a Z-score greater than or equal to 3. The substrates were then validated using the standard solution protein kinase-substrate phosphorylation assay. Twenty-three out of the thirty-two substrates were validated by having signals greater than or equal to three times the background (the kinase treated band over the non-treated). Click here for file

Additional file 2 **Solution assay validation of protein phosphorylation defined through pooling deconvolution on protein microarrays**. Four protein kinase pools were prepared to probe Human ProtoArray^® ^in triplicate. Each kinase was also used to probe Human ProtoArray^® ^individually. Signals for each protein kinase substrate were Z-score transformed The average Z-score from three arrays probed with each pool plus the standard deviation (STDEV) of the signals are shown. For single kinase probing, only the Z-scores are shown. Z-scores greater than or equal to 3.0 are considered significant (positive) signals. Click here for file
