# Supplementary figures and images for: Protein kinase substrate identification on functional protein arrays
Source: BMC Biotechnol. 2008 Feb 28;8:22. doi: 10.1186/1472-6750-8-22 (PMC2270825; doi:10.1186/1472-6750-8-22)

## Slide 1
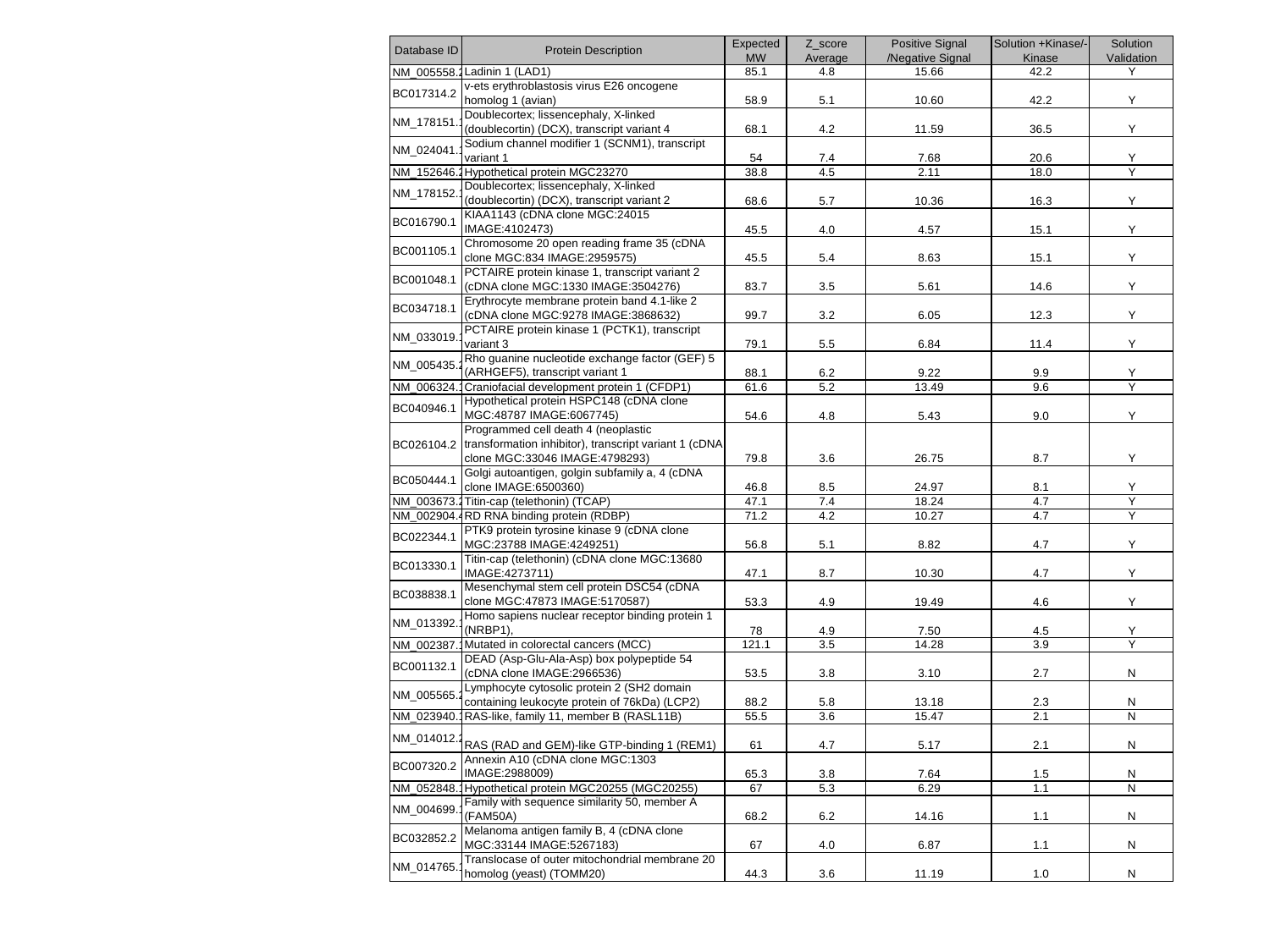

Supplement: Additional file 1 — CAMK II substrate identification. CamK II assays were performed on Human ProtoArrays® using three protocols – Buffer System I protocol, Buffer System II protocol, and lambda protein phosphatase-pretreatment protocol. Each assay condition was performed in triplicate, including the ATP negative controls. Substrates were identified by having a Z-score greater than or equal to 3. The substrates were then validated using the standard solution protein kinase-substrate phosphorylation assay. Twenty-three out of the thirty-two substrates were validated by having signals greater than or equal to three times the background (the kinase treated band over the non-treated). [file 1472-6750-8-22-S1.ppt]

## Slide 1
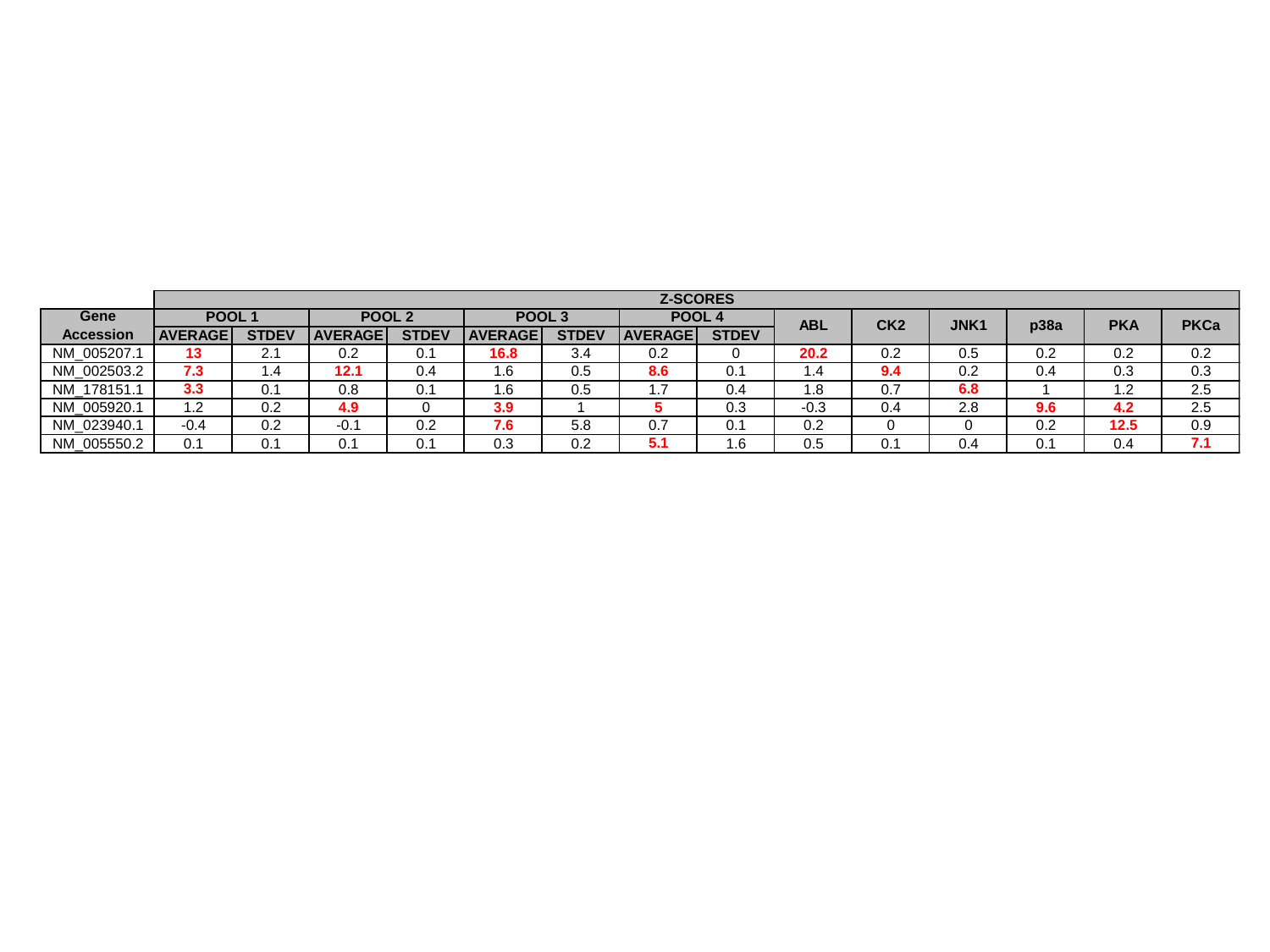

Supplement: Additional file 2 — Solution assay validation of protein phosphorylation defined through pooling deconvolution on protein microarrays. Four protein kinase pools were prepared to probe Human ProtoArray® in triplicate. Each kinase was also used to probe Human ProtoArray® individually. Signals for each protein kinase substrate were Z-score transformed The average Z-score from three arrays probed with each pool plus the standard deviation (STDEV) of the signals are shown. For single kinase probing, only the Z-scores are shown. Z-scores greater than or equal to 3.0 are considered significant (positive) signals. [file 1472-6750-8-22-S2.ppt]
